# Backward cloud transformation algorithm based on Kullback Leibler divergence

**DOI:** 10.1371/journal.pone.0341268

**Published:** 2026-01-27

**Authors:** Xiaobin Xu, Kangwei Yu, Junhe Fu, Lingjun Dong, Haohao Guo, Hong He, Lu Zhang

**Affiliations:** 1 China-Austria Belt and Road Joint Laboratory on Artificial Intelligence and Advanced Manufacturing, Hangzhou Dianzi University, Hangzhou, China; 2 School of Automation, Hangzhou Dianzi University, Hangzhou, Zhejiang, China; 3 Taizhou Special Equipment Inspection and Testing Research Institute, Taizhou, Zhejiang, China; 4 School of Communication Engineering, Hangzhou Dianzi University, Hangzhou, Zhejiang, China; 5 Colledge of Electronical and Information Engineering, QuZhou University, Qu Zhou, China; University of Tartu, ESTONIA

## Abstract

As a bidirectional cognitive model for dealing with uncertainty, cloud model (CM) are commonly used in application scenarios such as fault diagnosis, system modeling, and evaluation. CM achieves bidirectional conversion between qualitative concepts and quantitative values through forward cloud transformation (FCT) and backward cloud transformation (BCT) algorithms. Among them, the BCT algorithm obtains key parameters that characterize the randomness and fuzziness of concepts through the analysis of quantitative sample data, namely the expectation (*Ex*), entropy (*En*), and hyper entropy (*He*) for CM. However, the existing BCT algorithms adopt an integrated modeling approach, ignoring the impact of data with different distribution characteristics on obtaining key parameters for CM. Therefore, this paper proposes a BCT algorithm based on Kullback Leibler (KL) divergence, aiming to refine the process for obtaining key parameters of CM by analyzing the distribution differences between sample data. Firstly, the corresponding atomization template dataset (ATD) is obtained by conducting a coarse-grained analysis of the sample data. Then, calculate the KL divergence between sample data and ATD to effectively evaluate the atomization state of CM after transforming the sample data. Based on the evaluation results, two differentiated BCT strategies are designed for both atomization and non-atomization states to obtain key parameters of CM. Finally, a comparative analysis is conducted between the proposed method and traditional BCT algorithms, using the University of California Irvine (UCI) benchmark dataset and real fault diagnosis data for error analysis. The experimental results indicate that the proposed method can obtain more accurate key parameters of CM than other BCT algorithms.

## 1. Introduction

Sensor monitoring often faces data from different sources, which have complex and variable characteristics. To effectively process and analyze these data, we need to rely on a series of mathematical and statistical methods [1–3]. Among these methods, probability theory and fuzzy set theory are two very important tools that provide modeling and analysis frameworks for different characteristics of data. Probability theory is a mathematical model based on the assumption of randomness, which describes the uncertainty of data through probability distributions [4, 5]. The core of this theory lies in its ability to quantify the probabilities of various events that may occur in data, thereby predicting the trend of data changes. For example, in the financial field, by statistically modeling the collected financial data and using Monte Carlo simulation methods to generate a large number of possible future scenarios, risk monitoring personnel can assess potential investment risks and return distributions. This method can provide a scientific basis for decision-making by repeatedly sampling and calculating the performance of different investment portfolios under different market conditions [6,7]. However, probability theory typically assumes that the uncertainty of data is random. In practice, the data we face not only has randomness but also fuzziness. Fuzziness may hinder the representation of data using precise numerical values or clear classifications [8]. For example, in the medical field, patients’ pain perception is often difficult to accurately describe with a single numerical value. Pain is a subjective experience that is influenced by various factors, including an individual’s pain threshold, emotional state, and specific pathological conditions. To better express the intensity of pain, fuzzy logic models are often used to quantify patients’ pain levels, which can more comprehensively capture and represent the uncertainty in pain data [9].

Fuzzy set theory provides a solution to the fuzziness of such data. It uses fuzzy logic to handle uncertainty and fuzziness in data, allowing for transitions and overlaps between different categories [10,11]. The core of fuzzy set theory is the concept of fuzzy sets, which allows elements to belong to sets to different degrees, rather than the “all or nothing” binary logic in traditional set theory [12]. This theory is particularly useful in handling qualitative data, expert systems, and decision support systems [13,14]. However, when data has both fuzziness and randomness, a single probability model or fuzzy model makes it difficult to fully capture the characteristics of the data. To address this issue, Professor Deyi Li proposed the cloud model (CM) [15,16]. CM constructs a data model that can simultaneously describe randomness and fuzziness by organically combining the three parameters of expectation (*Ex*), entropy (*En*), and hyper entropy (*He*). This model not only captures the central tendency of data but also describes the degree of data dispersion and fuzzy boundaries, thereby improving the model’s expressive power and adaptability. Among them, *Ex* is the most representative point in quantitative data, and the closer the data is to *Ex*, the more concentrated the distribution; On the contrary, the further away from *Ex*, the more dispersed the distribution. *En* is a first-order measure of data uncertainty that reflects the overall range of data points. And *He* is a high-order measure of *En*, which is determined by the randomness and fuzziness of *En*. The larger the *He*, the greater the thickness of CM constructed from the data.

The CM provides a new solution for handling data with randomness and fuzziness. By uniformly representing randomness and fuzziness, the model can better adapt to complex and ever-changing practical problems. In practical applications, CM has been widely used in multiple fields, such as image processing, fault diagnosis of industrial equipment, and natural disaster prediction [17–19]. For example, Li et al. divided the histogram of the input image into several intervals and used CM to achieve multimodal fusion and enhancement for multiple different images [20]. Xu et al. adopted a method based on the normal cloud model to characterize data containing fault feature information and generate interval-valued evidence that describes the occurrence of faults. Furthermore, by integrating diagnostic evidence provided by multiple information sources, the reliability and accuracy of motor rotor fault diagnosis results have been improved [21]. Lin et al. used the rough set (RS) theory to determine the weights of indicators that affect rock bursts in the sample data and used CM to classify and evaluate rock bursts. The constructed RS-CM model achieved higher-precision prediction of rock burst occurrence [22].

In the process of CM modeling, the characteristics of the modeled data mainly depend on three key parameters (*Ex*, *En*, and *He*). Therefore, it is crucial to accurately obtain these parameters. The traditional parameter determination methods mainly rely on manual setting or numerical calculation. The former is subject to the limitations of human subjectivity and insufficient information, leading to insufficient accuracy in the parameters obtained. In contrast, numerical calculations, especially BCT algorithms, are widely used. Liu et al. proposed a Backward Cloud Algorithm based on the First Order Absolute Central Moment (SBCT-1thM). The SBCT-1thM algorithm determines the key parameters of CM through the data itself, without relying on the certainty degree of the data. This method has received widespread attention for its simplicity and practicality [23]. However, for highly concentrated data, the variance of the data is very small, and there may be situations where the estimated variance is smaller than the estimated *En*. In this case, the obtained *He* is a complex number, indicating an invalid modeling. Wang [24] et al. further proposed the Backward Cloud Algorithm based on the Fourth Order Absolute Central Moment (SBCT-4thM), which estimates *En* values through the fourth-moment of the data. Although this method has certain innovations in theory, it is also difficult to completely avoid this complex number phenomenon in practical applications. On this basis, Wang [25,26] et al. designed a Multiple Backward Cloud Transformation based on Sampling with Replacement (MBCT-SR) algorithm, which first samples and groups the original data, utilizing the obtained mean and variance of multiple samples to calculate *En* and *He*, thereby effectively avoiding complex number phenomena. However, due to the unified modeling approach adopted by the algorithm, it is difficult to achieve ideal accuracy for data with different distribution characteristics.

When constructing CM, for data with different distribution characteristics, the generated cloud graphs can be divided into two types: non-atomization and atomization. When in the atomization state, the cloud drops in the CM appear in a discrete state and the cohesiveness of the cloud drops is low. This phenomenon reflects that at this time, the sample data cannot form a consistent understanding of the inherent characteristics for the data, namely the meaning represented by these data cannot be accurately understood [27,28]. Accurately evaluating the atomization degree is a prerequisite for the next step of refinement to obtain En and *He*. Therefore, this paper proposes a CM construction method based on KL divergence, aiming to refine the process for obtaining key parameters of CM by quantitatively analyzing the distribution differences between sample data. Firstly, by calculating the KL divergence between different sample data, the distribution structure relationship of the data can be effectively analyzed. KL divergence, as a measure of probability distribution differences, can reveal the inherent distribution characteristics for sample data. Then, based on the analysis results of KL divergence, predict the transformed CM state of the sample data, and make a reasonable decision on whether the data is in a non-atomization or atomization state. After evaluating the atomization degree, we further design a differentiated transformation strategy to obtain key parameters of CM. This method considers the data characteristics under different atomization degrees. Compared with traditional BCT algorithms, our method models the data more finely, revealing the distribution relationship between data more comprehensively and improving the accuracy and stability of the model.

The remaining parts of this paper are arranged as follows. Section 2 mainly introduces some basic theoretical knowledge, including the definitions of CM and normal cloud model (NCM), as well as the concept of KL divergence. Section 3 proposes a BCT algorithm based on KL divergence, introduces how to use KL divergence values to evaluate the atomization state for the data to be modeled (DBM), and provides BCT strategies for different atomization states. In Section 4, the effectiveness of the proposed method was validated through modeling examples of simulated and actual data for motor rotor fault monitoring, and a cost analysis of the method was conducted. Finally, the conclusion is presented in Section 5.

## 2. Relevant theoretical foundations

### 2.1. Normal cloud theory

**Definition 1.** Cloud Model. The qualitative concept *C* is defined on the quantitative universe *U*. If x∈U is a random implementation of concept *C*, and the certainty degree μ(x)∈[0,1] of *x* concerning *C* is a random number with stable distribution: μ(x):U→[0,1]∀x∈U, then the distribution of *x* on the universe *U* is called a cloud model, and *x* is called a cloud drop.

**Fig 1:**
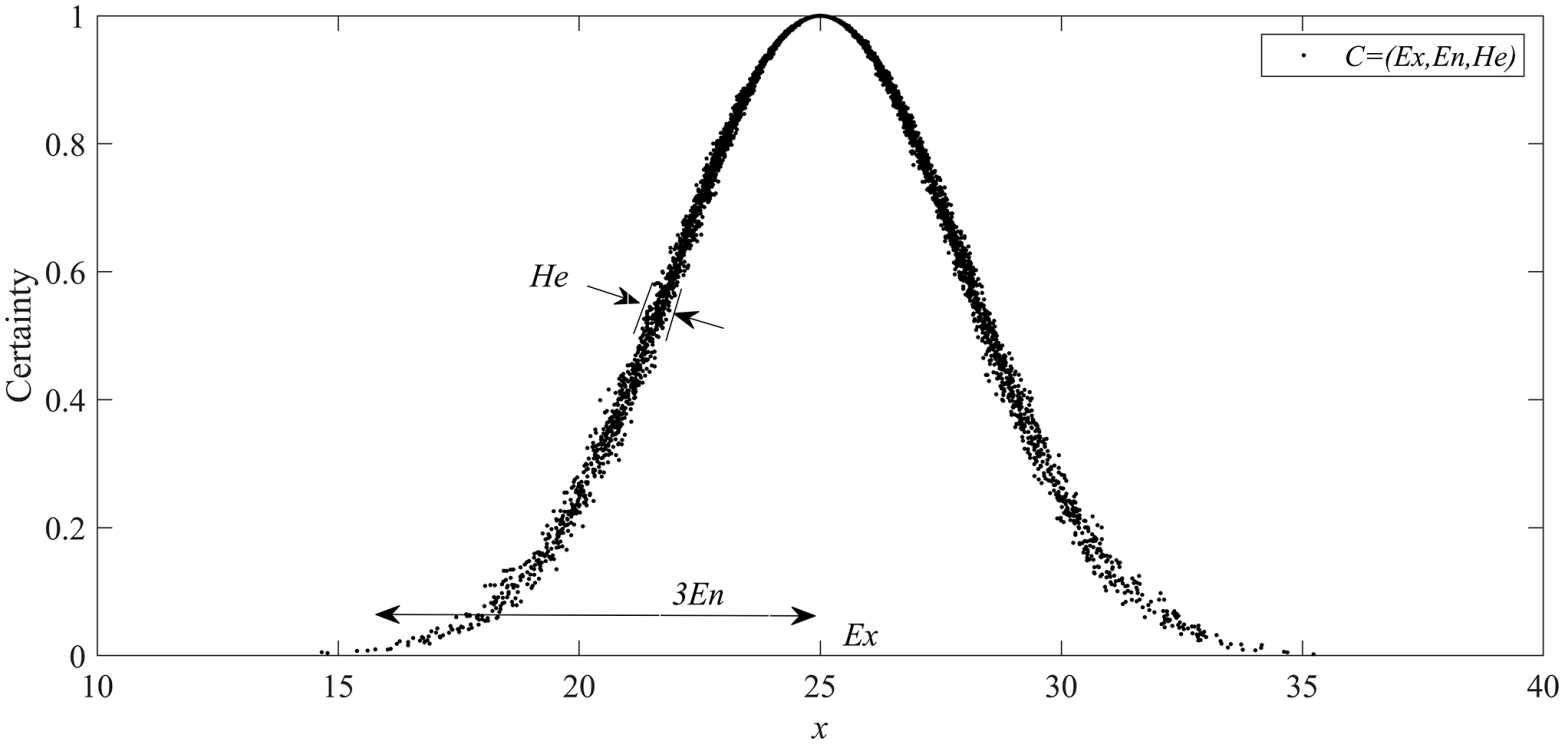
The normal cloud model.

**Definition 2.** Normal Cloud Model. The qualitative concept *C* is defined on the quantitative universe *U*, and *C* contains three key parameters (*Ex*, *En*, *He*). Suppose *x* is distributed as a cloud model on the quantitative universe *U*, where cloud drop *x* satisfies $x\sim 𝒩(Ex,{\left|y\right|}^{2}),y\sim 𝒩(En,He^{2})$ and the certainty degree function satisfies $\mu (x)=\exp(-\frac{{\left(x-Ex\right)}^{2}}{2y^{2}})$. In that case, the distribution of the random variable *X* composed of all cloud drop *x* is called a normal cloud model, denoted as *C* = (*Ex*, *En*, *He*). This is illustrated specifically in Fig 1.

It has been shown through extensive research that the objective changes in most qualitative concepts follow a normal distribution, making the NCM widely applicable in data modeling [29,30].

**Definition 3.** The Key Parameters of the Cloud Model. Expectation (*Ex*): It is the central value of the distribution for cloud drops in *U*, which is also the most representative data point for *C*, or in other words, the value that reflects the most likely occurrence of *C*. When it comes to a given concept, *Ex* often represents the numerical typicality of that concept. For all cloud drops, the closer they are to *Ex*, the more concentrated they are; the farther they are from *Ex*, the more scattered and sparse the cloud drops are.

Entropy (*En*): It is a measure of discreteness or fuzziness for CM, which describes the quantitative uncertainty of *C* and reflects the width of its entire numerical range in the *U*. Furthermore, it is determined by the randomness and fuzziness of *C*. On the one hand, *En* represents the degree of fuzziness for *C*, that is, the quantitative scalability of *C*; On the other hand, it also reflects the uncertainty or range of change for *C*, indicating the possible fluctuations of *C* in different situations. The larger the *En*, the greater the fluctuation range of *C* in different situations and the higher the uncertainty.

Hyper entropy (*He*): It is the entropy of *En*, that is, the degree of dispersion for *En*. It reflects the degree of stability for the uncertainty of *C*. The larger *He*, the greater the change in *En*, indicating that the greater the fuzziness and uncertainty of *C* in different situations. *He* represents the range of uncertainty in a concept and reflects the stability of *En* itself.

**Definition 4.** FCT and BCT for CM. The forward cloud transformation (FCT) for CM refers to the process of generating quantitative cloud drops *x* through the known key parameters (*Ex*, *En*, *He*). In this process, *x* is a normal random number generated with *Ex* as the expectation and |y| as the standard deviation, where *y* is a normal random number generated with *En* as the expectation and *He* as the standard deviation.

**Fig 2:**
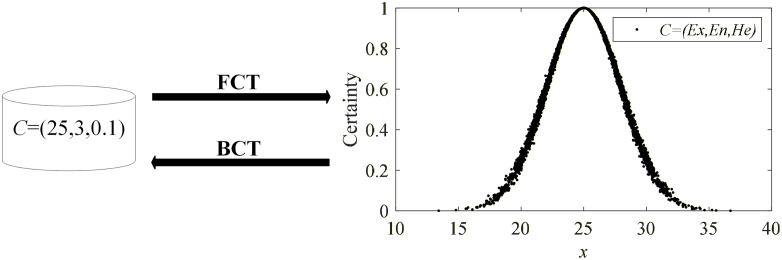
Forward and backward cloud transformation processes.

The backward cloud transformation (BCT) for CM refers to the process of estimating key parameters (*Ex*, *En*, *He*) through a set of known quantitative cloud drops. This process involves analyzing the distribution and statistical characteristics for cloud drops to infer the parameters of CM that generated them. The process of FCT and BCT are shown in Fig 2.

**Definition 5.** Statistical Properties of Normal Cloud Model

According to Definition 2, all random variables *Y* composed of *y* follow a normal distribution with *En* as the expectation and *He* as the standard deviation. Therefore, the probability density function of *Y* satisfies Eq. (1).


$$f_{Y}(y)=\frac{\text{1}}{\sqrt{2\pi }He}e^{-\frac{{\left(y-En\right)}^{2}}{2He^{2}}}$$ (1)

When the random variable *Y* = *y* is constant, the random variable *X* follows a normal distribution with *Ex* as the expectation and standard deviation, so the conditional probability density function of the random variable *X* satisfies Eq. (2).


$$f_{X|Y}(x|Y=y)=e^{-\frac{{\left(x-Ex\right)}^{2}}{2y^{2}}}$$ (2)

According to Eq. (3), we can obtain the probability density function of the random variable *X* as shown in Eq. (4).


$$f_{X,Y}(x,y)=f_{X|Y}(x|Y=y)f_{Y}(y)$$ (3)

$$f_{X}(x)=\int_{-\infty }^{+\infty }f_{X,Y}(x,y)dy=\frac{\text{1}}{2\pi He}\int_{-\infty }^{+\infty }\frac{\text{1}}{\left|y\right|}e^{-\frac{{\left(x-Ex\right)}^{2}}{2y^{2}}-\frac{{\left(y-En\right)}^{2}}{2He^{2}}}$$ (4)

Especially when *He* = 0, the probability density function of the random variable *X* is the density function of the normal distribution $𝒩(En,He^{2})$. By numerically integrating the probability density function of the random variable *X*, the following important statistical properties can be obtained [31,32]:

1. The mathematical expectation of second-order normal cloud *X* satisfies *EX* = *Ex*.
2. The variance of second-order normal cloud *X* satisfies *DX* = *En*^2^ + *He*^2^.
3. The third-order center moment of the second-order normal cloud *X* satisfies *E*(*X*-*Ex*)=0.
4. The fourth central moment of the second-order normal cloud *X* satisfies *E*(*X*-*EX*)^4^ = 9*He*^2^+18*En*^2^*He*^2^+3*En*^4^.

### 2.2. KL divergence

**Definition 6.** KL divergence. KL divergence, also known as relative entropy, is a method for describing the difference between two probability distributions *P* and *Q*. It can be used to measure the degree to which one distribution deviates from the other. If two distributions are the same, the divergence between them is 0. The smaller the KL divergence, the closer the two distributions are. Firstly, assuming two discrete probability distributions are *P* and *Q*, their KL divergence is as follow.


$$KL(P||Q)=\sum_{\alpha }P(\alpha )\log\frac{P(\alpha )}{Q(\alpha )}$$ (5)

where P(α) is the probability of event α under the distribution *P*, Q(α) is the probability of event α under the distribution *Q*.

When two distributions are normal, KL divergence calculation can be simplified as calculating through the parameters of the two normal distributions. If two normal distributions are $𝒩(\mu_{1},\sigma_{1}^{2})$ and $𝒩(\mu_{2},\sigma_{2}^{2})$, the calculation formula for their KL divergence can be defined by Eq. (6).


$$KL(P||Q)=\ln(\frac{\sigma_{2}}{\sigma_{1}})+\frac{\sigma_{1}^{2}+{\left(\mu_{1}-\mu_{2}\right)}^{2}}{2\sigma_{2}^{2}}-\frac{\text{1}}{\text{2}}$$ (6)

where *P* represents the reference distribution, and *Q* represents the target distribution.

## 3. KL divergence-based sampling with replacement BCT

Considering the universality of NCM, the cloud models constructed in this paper all adopt the form of NCM. The traditional BCT method conducts an integrated analysis for data, neglecting the impact of data with different distribution characteristics on obtaining CMKP. Therefore, this paper proposes a more refined processing method aimed at accurately extracting CMKP. In this process, we introduce KL divergence to quantitatively evaluate the data atomization degree and adopt different BCT strategies based on the atomization degree. This section will provide a detailed introduction to the complete process of the BCT based on KL divergence. Section 3.1 presents the overall framework of Kullback-Leibler Divergence-based Sampling with Replacement Backward Cloud Transformation Algorithm (KL-SR). Section 3.2 provides a detailed introduction to the process of estimating CM atomization degrees, and Section 3.3 proposes different BCT strategies for modeling data with different atomization degrees to achieve accurate estimation of CMKP.

### 3.1. KL-SR algorithm framework

**Fig 3:**
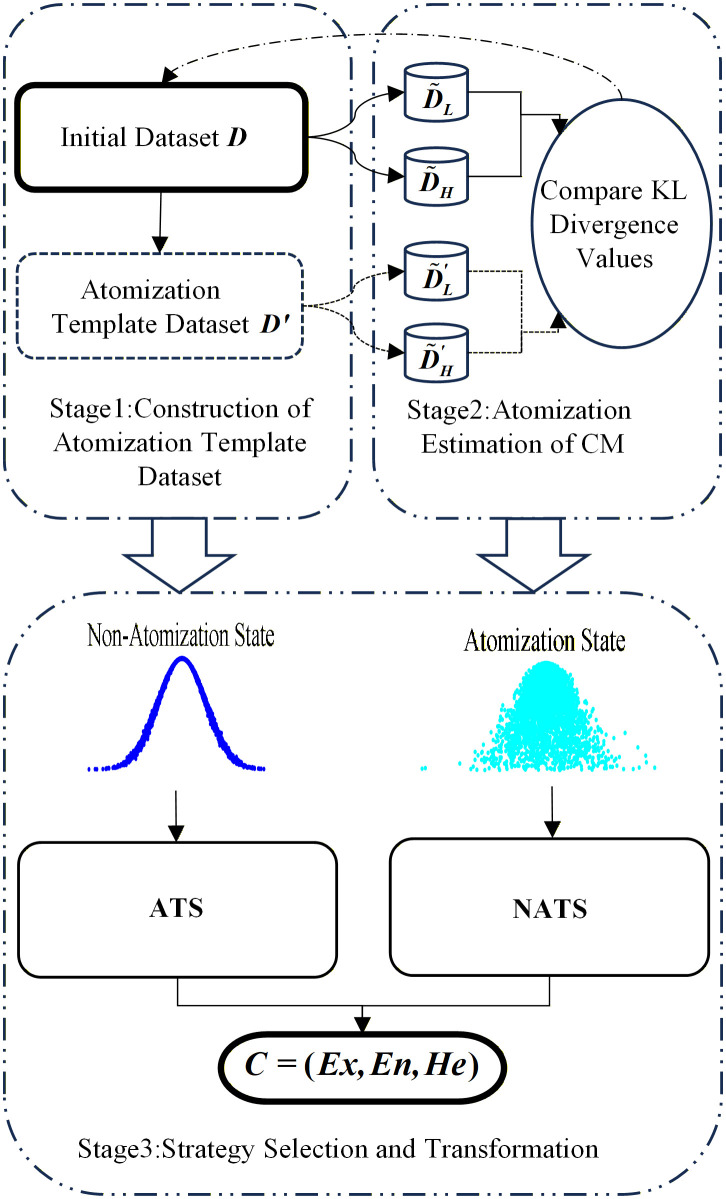
Overall framework for KL-SR.

To obtain a more accurate CMKP, the BCT algorithm proposed in this paper utilizes KL divergence to measure the internal distribution structure relationship of the data, and based on this, determines the atomization state of the constructed CM. We adopt a parameter estimation strategy to enhance the accuracy of BCT for the data to be modeled that was judged to be in an atomization state; For non-atomization state data to be modeled, we improve the accuracy of BCT by analyzing the statistical characteristics for the data. The framework of the entire algorithm is shown in Fig 3.

From Fig 3, it can be seen that the KL-SR algorithm can be divided into three main stages:

1. Construction of atomization template dataset. By conducting coarse-grained analysis on the modeling data, obtain the key parameters of the atomization template cloud model (ATCM). Based on this, the FCT method is used to obtain the atomization template dataset.
2. Atomization estimation of CM. By utilizing KL divergence to quantify the internal distribution characteristics of data and comparing it with the KL divergence of data in the atomization template dataset, the atomization state of the data to be modeled can be estimated.
3. Strategy selection and transformation. Based on the estimated atomization degree obtained from the previous analysis, appropriate BCT strategies are adopted for different atomization states to improve the accuracy of CMKP.

In the entire process of the KL-SR algorithm, the atomization estimation of CM and strategy selection and transformation are the core of the algorithm. By finely processing and analyzing modeling data, and designing differentiated strategies for different atomization states, the accuracy and reliability of obtaining CMKP through BCT can be improved. The subsequent sections of this paper will detail these critical steps.

### 3.2. Atomization estimation of CM

**Fig 4:**
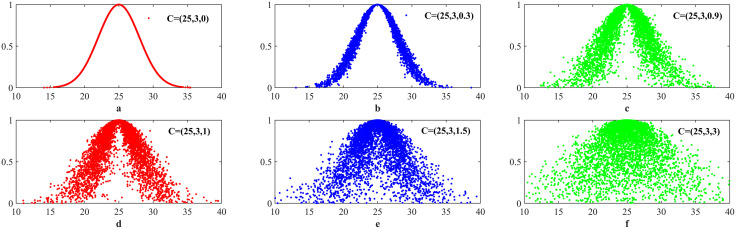
Cloud map constructed from data of atomization and non-atomization states.

Due to the different distribution structures of various data, there are also differences in the cloud maps generated by the CM. This difference is not only reflected in the intuitive visual representation of the cloud map but also in CMKP. Specifically, for the second-order NCM, when the *He* = 0, the second-order normal cloud will degenerate into a normal distribution; When the *He* increases to 0 ≤ *He* ≤ *En*/3, the cloud drops gradually disperses and the cohesiveness weakens; When the *He* further increases to *He* ≥ *En*/3, the second-order normal cloud exhibits an atomization state. Fig 4 shows the cloud maps generated at different *En* and *He* ratios, where *a*, *b*, and *c* are the cloud maps corresponding to non-atomization state. It can be seen that the cloud drop distribution of the NCM shows a clear concentration trend, and the distribution range of cloud drops is relatively narrow. At this time, the *He* is relatively small, and the cloud drops mainly gather near *Ex*, forming a relatively clear cloud shape. In Fig 4, *e* and *f* are cloud maps corresponding to the atomization state, which show that as the *He* increases, the distribution of cloud drops begins to become more dispersed, and CM transitions from cloud to fog, namely the distribution of cloud drops in the numerical domain space becomes more extensive and fuzzier. And *d* is the critical cloud map, in which the distribution of cloud drops exhibits a transitional characteristic, with both the concentration and dispersion of cloud drops at an intermediate level.

To accurately measure the atomization degree of the data to be modeled, we determine it by analyzing the KL divergence difference between it and the atomization template dataset. For a set of data $D=(x_{1},x_{2},...,x_{n},...,x_{N})$ to be modeled, first perform coarse-grained analysis on it, construct a corresponding ATCM, and use the cloud drops in the model as the atomization template dataset. The specific steps are as follows.

Stage 1.1: According to the data *D* to be modeled, the cloud model key parameters (CMKP) can be obtained through Eqs. (7)–(9).


$$Ex^{\prime }=\frac{x_{1}+x_{2}+...+x_{N}}{N}$$ (7)

$$En^{\prime }=\sqrt{\frac{{\left(x_{1}-Ex^{\prime }\right)}^{2}+{\left(x_{2}-Ex^{\prime }\right)}^{2}+...+{\left(x_{N}-Ex^{\prime }\right)}^{2}}{N-1}}$$ (8)

$$He^{\prime }=\frac{En^{\prime }}{\text{3}}$$ (9)

**Remark 1:** In order to verify the rationality of *He* = *En*/3 in the cloud model parameters of the atomization template, we designed multiple comparative experiments and evaluated their impact on cloud model modeling of real data. The specific experimental setup, result data, and analysis are detailed in the S1 File.

Stage 1.2: Using $En^{\prime }$ as the expectation and $He^{\prime }$ as the standard deviation, generate a normal random number $y_{m}$ that satisfies $y_{m}\sim 𝒩(En^{\prime },He^{2})$. Then, using $Ex^{\prime }$ as the expectation and $\left|y_{m}\right|$ as the standard deviation, generate another normal random number ${𝒳}_{m}$ that satisfies ${𝒳}_{m}\sim 𝒩(Ex,{\left|y_{m}\right|}^{2})$.

Stage 1.3: Repeat Stage 1.2 *M* times until *M* normal random numbers are generated to form the atomization template dataset $D^{\prime }=({𝒳}_{1},{𝒳}_{2},...,{𝒳}_{m},...,{𝒳}_{M})$, where *M* = 5000.

**Remark 2:** In the implementation of the KL-SR algorithm, both the initial number of input cloud drops and the size of the atomization template set are set to 5000. This setting is primarily motivated by two considerations: first, to ensure sufficient statistical stability for parameter estimation; and second, to strike a practical balance between computational efficiency and estimation accuracy. To systematically validate the rationale behind this parameter choice, a dedicated comparative experiment was designed. The detailed experimental design and analysis are provided in S2 File.

By following the above steps, the atomization template dataset generated based on the data *D* to be modeled can be obtained. To further measure the distribution structure of *D*, it is necessary to calculate the KL divergence values of the low and high-order subsets corresponding to *D* and $D^{\prime }$ respectively, to estimate the atomization degree of *D*. The specific steps are as follows.

Stage 2.1: Sort the *M* cloud drops in the $D^{\prime }$ in ascending order of their numerical values to obtain the sorted data sequence ${\tilde{D}}^{\prime }=({\tilde{𝒳}}_{1},{\tilde{𝒳}}_{2},...,{\tilde{𝒳}}_{m},...,{\tilde{𝒳}}_{M})$, which satisfies ${\tilde{𝒳}}_{1}<{\tilde{𝒳}}_{2}<...<{\tilde{𝒳}}_{M}$;

Stage 2.2: Divide the sorted data sequence ${\tilde{D}}^{\prime }$ evenly into two parts, namely the low-order subset ${\tilde{D}}_{L}^{\prime }$ and the high-order subset ${\tilde{D}}_{H}^{\prime }$, where ${\tilde{D}}_{L}^{\prime }=({\tilde{𝒳}}_{1},{\tilde{𝒳}}_{2},...,{\tilde{𝒳}}_{\frac{M}{\text{2}}})$, ${\tilde{D}}_{H}^{\prime }=({\tilde{𝒳}}_{\frac{M}{\text{2}}+1},{\tilde{𝒳}}_{\frac{M}{\text{2}}+2},...,{\tilde{𝒳}}_{M})$;

Stage 2.3: Similarly, for the data *D* to be modeled, based on steps Stage 2.1 and Stage 2.2, a low-order subset ${\tilde{D}}_{L}=({\tilde{x}}_{1},{\tilde{x}}_{2},...,{\tilde{x}}_{\frac{N}{\text{2}}})$ and a high-order subset ${\tilde{D}}_{H}=({\tilde{x}}_{\frac{N}{\text{2}}+1},{\tilde{x}}_{\frac{N}{\text{2}}+2},...,{\tilde{x}}_{N})$ can be obtained sequentially, where ${\tilde{x}}_{1}<{\tilde{x}}_{2}<...<{\tilde{x}}_{N}$. If *N* is odd, the middle elements are assigned to either group.

Stage 2.4: Based on the ${\tilde{D}}_{L}$ and ${\tilde{D}}_{H}$ obtained in Stage 2.3, calculate their means $\mu_{L}$ and $\mu_{H}$ and standard deviations $\sigma_{L}$ and $\sigma_{H}$, respectively. The KL divergence value $KL({\tilde{D}}_{L}||{\tilde{D}}_{H})$ between ${\tilde{D}}_{L}$ and ${\tilde{D}}_{H}$ can be obtained using Eq. (10).


$$KL({\tilde{D}}_{L}||{\tilde{D}}_{H})=\ln(\frac{\sigma_{H}}{\sigma_{L}})+\frac{{\sigma_{L}}^{2}+{\left(\mu_{L}-\mu_{H}\right)}^{2}}{2{\sigma_{H}}^{2}}-\frac{\text{1}}{\text{2}}$$ (10)

For ${\tilde{D}}^{\prime }$, use the same method to calculate the KL divergence value $KL({\tilde{D}}_{L}^{\prime }||{\tilde{D}}_{H}^{\prime })$ of ${\tilde{D}}_{L}^{\prime }$ and ${\tilde{D}}_{H}^{\prime }$.


$$KL({\tilde{D}}_{L}^{\prime }||{\tilde{D}}_{H}^{\prime })=\ln(\frac{\sigma_{H}^{\prime }}{\sigma_{L}^{\prime }})+\frac{\sigma {L\prime }^{2}+{\left(\mu_{L}^{\prime }-\mu_{H}^{\prime }\right)}^{2}}{2\sigma {H\prime }^{2}}-\frac{\text{1}}{\text{2}}$$ (11)

Stage 2.5: Determine the atomization state of *D* based on the atomization decision criteria shown in Eq. (12).


$$D=\left\{ \begin{array}{c} Non-Atomization\;State\;if\;KL({\tilde{D}}_{L}||{\tilde{D}}_{H})>KL({\tilde{D}}_{L}^{\prime }||{\tilde{D}}_{H}^{\prime })\\ Atomization\;State\;if\;KL({\tilde{D}}_{L}||{\tilde{D}}_{H})<KL({\tilde{D}}_{L}^{\prime }||{\tilde{D}}_{H}^{\prime }) \end{array}\right.$$ (12)

Based on the above steps, by estimating the atomization degree of the *D*, a foundation is laid for selecting appropriate BCT strategies under different atomization degrees.

### 3.3. Strategy selection and transformation

To accurately obtain CMKP, we design two BCT strategies, namely the Non-Atomization Backward Cloud Transformation Strategy (NATS) and the Atomization Backward Cloud Transformation Strategy (ATS). NATS mainly targets the data to be modeled that is judged to be in a non-atomization state, which has low uncertainty and the atomization degree of the generated cloud map is not obvious. Conversely, ATS focuses on processing the data to be modeled that is judged to be in an atomization state. In this case, the uncertainty of the data is high, and the atomization phenomenon of the generated cloud map is significant. By adopting a differentiated approach to obtain CMKP of the data to be modeled, effective estimation can be achieved.

Non-Atomization Backward Cloud Transformation Strategy (NATS): For the data to be modeled that is judged to be in a non-atomization state, the generated cloud map tends towards the states shown in *a*, *b*, and *c* from Fig 4. In the non-atomization state, the cloud map corresponding to the data *D* to be modeled shows relative concentration and normal distribution characteristics.

According to the definition 2 of the normal cloud in Section 2, cloud drop *x* satisfies $x=𝒩(Ex,{\left|y\right|}^{2}),y=𝒩(En,He^{2})$. Therefore, the normal random number *y* follows a normal distribution with a mean of *En* and a standard deviation of *He*. According to the “3*He*” principle of normal distribution, in the process of generating quantitative cloud drops through FCT, about 99.74% of the normal random number *y* is located in the interval [*En*-3*He*, *En* + 3*He*] [33,34]. From the certainty degree function corresponding to cloud drop *x* in definition 2, it can be seen that when 0 < *He* < *En*/3, 99.74% of the cloud drop’s certainty degree will be distributed between the inner and outer contour of CM. The inner domain contour and outer domain contour are shown in Eqs. (13) and (14).


$$\mu_{1}(x)=\exp\left(-\frac{{\left(x-Ex\right)}^{2}}{2{\left(\sigma_{IE}\right)}^{2}}\right)$$ (13)

$$\mu_{2}(x)=\exp\left(-\frac{{\left(x-Ex\right)}^{2}}{2{\left(\sigma_{OE}\right)}^{2}}\right)$$ (14)

where $\sigma_{IE}=En-3He$ is the inner domain feature parameter, and $\sigma_{OE}=En+3He$ represents the outer domain feature parameter.

Therefore, based on the analysis of the certainty degree distribution for cloud drops in non-atomization states, we propose a BCT strategy for the data to be modeled in non-atomization states. The specific process is as follows.

Stage 3.1: For the data $D=(x_{1},x_{2},...,x_{n},...,x_{N})$ to be modeled, take their mean as the *Ex*.


$$Ex=\frac{x_{1}+x_{2}+...+x_{N}}{N}$$ (15)

Stage 3.2: Randomly group the sample data $x_{1},x_{2},...,x_{N}$ in *D* into *S* groups, with a total of $n_{s}$ for data in each group. The sample assigned to the *s*th group can be represented as $X^{s}=\{x_{s}(1),x_{s}(2),...,x_{s}(n_{s}\}$. The number of groups *S* and the number of elements in each group $n_{s}$ can be acquired from Eqs. (16) and (17), respectively.


$$S=1+\frac{\ln(M)}{\ln(2)}$$ (16)

$$n_{s}=\frac{M}{S}$$ (17)

Stage 3.3: For the *s*th sample $X^{s}=\{x_{s}(1),x_{s}(2),...,x_{s}(n_{s}\}$, calculate its expectation value $Ex_{s}$ and standard deviation $\sigma_{s}$ to obtain the set of expectation values $\left\{Ex_{1},Ex_{2},...,Ex_{S}\right\}$ and standard deviation $\left\{\sigma_{1},\sigma_{2},...,\sigma_{S}\right\}$ corresponding to the *S*-group of samples. Based on Eqs. (18) and (19), the feature parameters of the inner and outer domains can be derived.


$$\sigma_{IE}=\frac{\min\{Ex_{1}+3\sigma_{1},...,Ex_{S}+3\sigma_{S}\}-\max\{Ex_{1}-3\sigma_{1},...,Ex_{S}-3\sigma_{S}\}}{\text{6}}$$ (18)

$$\sigma_{OE}=\frac{\max\{Ex_{1}+3\sigma_{1},...,Ex_{S}+3\sigma_{S}\}-\min\{Ex_{1}-3\sigma_{1},...,Ex_{S}-3\sigma_{S}\}}{\text{6}}$$ (19)

Stage 3.4: According to the $\sigma_{IE}$ and $\sigma_{OE}$ obtained from Stage 3.3, we can calculate the *En* and *He* of CM. The specific calculation formula is as follows.


$$En=\frac{\sigma_{IE}+\sigma_{OE}}{\text{2}}$$ (20)

$$He=\frac{\sigma_{OE}-\sigma_{IE}}{\text{6}}$$ (21)

Atomization Backward Cloud Transformation Strategy (ATS): For the data to be modeled that is judged to be in an atomization state, the generated cloud map often approaches the states shown in *e* and *f* from Fig 4. In this state, the distribution of cloud drops that make up the cloud map is relatively discrete, and the *He* is much greater than *En*/3. For second-order normal clouds, when *He* > 0, the second-order normal cloud exhibits a peak state, and as *He* further increases, the distribution of cloud drops shows more pronounced peak characteristics.

**Fig 5:**
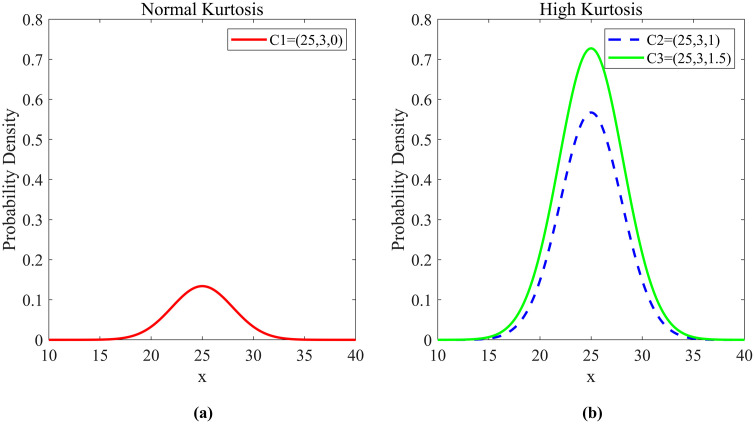
PDF of different kurtosis distributions.

To further demonstrate this phenomenon intuitively, we select three cloud models with different *En* and *He*, denoted as *C*1=(25,3,0), *C*2=(25,3,1), *C*3=(25,3,1.5) respectively. Meanwhile, we set 5000 cloud drops generated through FCT as the data to be modeled. For these datasets to be modeled with different distribution structures, we establish corresponding standardized probability density function (PDF) graphs, as specifically shown in Fig 5.

The left figure shows PDF graph mesokurtic characteristics, while the right figure reflects the PDF graph with leptokurtic characteristics. From Fig 5, it can be clearly seen that when the *He* is too large, the constructed PDF presents a leptokurtic state. As *He* gradually increases, the “heavy-tailed” phenomenon of the leptokurtic distribution becomes more pronounced. This feature can be described in statistics by the kurtosis coefficient, which measures the peakedness of a probability distribution and the concentration of its tails [35,36].

For the random variable *X*, if its fourth moment exists, the kurtosis coefficient *K* of the *X* is shown in Eq. (22).


$$K=\frac{E{\left(X-EX\right)}^{4}}{{\left(DX\right)}^{2}}-3$$ (22)

Due to the different distribution characteristics for different types of datasets, their corresponding kurtosis coefficients will also vary accordingly. The *K* of a normal distribution is 0, indicating that it has moderate peakedness and concentration, exhibiting common mesokurtic distribution characteristics. Compared to the normal distribution, a leptokurtic distribution is characterized by a sharp peak and concentrated tails, while a platykurtic distribution exhibits a flatter peak and more widely spread tails [37,38]. This demonstrates that changes in *He* have a significant impact on the kurtosis characteristics of cloud drop distribution, thereby reflecting the distribution features of the dataset.

For the dataset to be modeled in the atomization state, the corresponding *He* is relatively large, which usually indicates that its PDF is steeper based on the standard normal distribution, i.e., *K* > 0, showing a leptokurtic state. As *He* continues to increase, the leptokurtic state of the cloud drop distribution becomes more pronounced. Therefore, based on the analysis of the cloud drop distribution corresponding to the CM in an atomization state, we have designed a corresponding ATS for the data to be modeled that is judged as an atomization state. The specific process is as follows.

Stage 3.5: For the sample data in dataset $D=(x_{1},x_{2},...,x_{n},...,x_{N})$ to be modeled, set their mean as *Ex*.


$$Ex=\frac{x_{1}+x_{2}+...+x_{N}}{N}$$ (23)

Stage 3.6: Calculate the variance $q_{1}$ and fourth-order central moment $q_{2}$ of the *D*. Based on Eq. (22), let *r* = *He*/*En* to obtain the following equation.


$$K=\frac{q_{2}}{{\left(q_{1}\right)}^{2}}-3=6-6(1+r^{2}{)}^{-2}$$ (24)

where, $q_{1}=\frac{\text{1}}{N-1}\sum_{n=1}^{N}{\left(x_{n}-\bar{x}\right)}^{2}$, $q_{2}=\frac{\text{1}}{N}\sum_{n=1}^{N}{\left(x_{n}-\bar{x}\right)}^{4}$, and *K* represent the kurtosis coefficient of the *D*.

Stage 3.7: Based on Stage 3.2 and Stage 3.3, the external domain feature parameter $\sigma_{OE}$ can be obtained, and the *En* and *He* in CM can be calculate through Eqs. (25) and (26).


$$En=\frac{\sigma_{OE}}{1+3r}$$ (25)

$$He=\frac{r\cdot \sigma_{OE}}{1+3r}$$ (26)

By using the two different BCT strategies mentioned above, we can accurately obtain CMKP corresponding to the data to be modeled in different atomization states. The NATS is aimed at data with low uncertainty and unclear atomization degree. By analyzing the statistical characteristics of the data to be modeled, corresponding CMKP can be generated. The ATS focuses on processing data with high uncertainty and significant atomization phenomena. By combining parameter estimation with kurtosis calculation, it achieves accurate extraction for CMKP. These different BCT strategies enhances its adaptability to data with varying atomization degrees. Next, we will further demonstrate the effectiveness of the method proposed in this paper through comparative experiments.

## 4. Algorithm verification

In this section, to verify the effectiveness of the proposed KL-SR algorithm, three widely recognized BCT algorithms (SBCT-1thM, SBCT-4thM, and MBCT-SR) are selected as comparison benchmarks. By comparing and analyzing the key parameter errors of CMs before and after cloud transformation with KL-SR algorithm, as well as the estimation errors of real data before and after, the performance of KL-SR algorithm can be comprehensively evaluated. Specifically, the error of CMKP is quantified by calculating the Euclidean distance between the estimated CMKP and the initial CMKP. The estimation errors of real data are evaluated by calculating the Euclidean distance between the original real data and the estimated data after algorithm transformation. At the same time, the cost issues of KL-SR algorithm and other BCT algorithms were analyzed in section 4.3.

### 4.1. Error analysis of BCT algorithm based on CMKP

To verify the effectiveness of the method proposed in this paper, CMs with different atomization degrees are used to generate quantitative cloud drops as modeling data through FCT based on their CMKP. The specific key parameters information for CMs are shown in Table 1.

**Table 1:** The key parameters information for CMs.

Modeling data	C=(*Ex*, *En*, *He*)	Cloud drop count
*D*_1_	(25,3,0.10)	5000
*D*_2_	(25,3,0.55)	5000
*D*_3_	(25,3,0.80)	5000

After obtaining 5000 cloud droplets as the modeling data *D*, the corresponding CMKPs are estimated based on four different BCT algorithms, and multiple repeated experiments are conducted to ensure the reliability of the results. The experimental process of BCT based on KL-SR algorithm is divided into three parts.

1. **Construction of atomization template dataset.** According to the method described in Stage 1.1 of Section 3.2, a coarse-grained analysis is conducted on the modeling data *D*_1_, *D*_2_, and *D*_3_. Through Eqs. (7)–(9), the key parameters of ATCM as shown in Table 2 are obtained. Based on this, the atomization template datasets $D_{1}^{\prime }$, $D_{2}^{\prime }$, and $D_{3}^{\prime }$ can be acquired by Stage 1.2 to Stage 1.4.

**Table 2:** The key parameters of ATCM.

Modeling data	C=(*Ex*, *En*, *He*)
*D*_1_	(25.0499,9.2959,3.0986)
*D*_2_	(25.0135,9.1506,3.0502)
*D*_3_	(25.0100,0.6622,0.4165)

1. **Atomization estimation of CM.** According to the methods described in Stages 2.1 to 2.4 of Section 3.2, the internal distribution characteristics of *D*_1_, *D*_2_, and *D*_3_ are quantified. Then, compare the KL divergence values of $D_{1}^{\prime }$, $D_{2}^{\prime }$, and $D_{3}^{\prime }$ using Eq. (12) to estimate the atomization state of the modeling data. The specific KL divergence values and atomization state estimation results are shown in Table 3.

**Table 3:** Atomization state estimation results.

Modeling data	KL divergence of *D*	KL divergence of *D*’	Atomization state
*D*_1_	3.2443	2.8267	Non-Atomization
*D*_2_	3.3275	2.8003	Non-Atomization
*D*_3_	1.6804	2.6154	Atomization

1. **Strategy selection and transformation.** Based on the evaluation results, we estimate the atomization degree of modeling data and adopt the NATS for modeling data *D*_1_ and *D*_2_. While for modeling data *D*_3_, we chose the ATS. By Eqs. (15)–(26), the CMKPs are accurately estimated.

To ensure the reliability of the results, we conduct 50 independent BCT experiments and compare our method with three typical methods (MBCT-SR, SBCT-1thM, SBCT-4thM) used to obtain CMKP. After each experiment, we calculate the average, average absolute error (AAE), and mean square error (MSE) of the estimated CMKP obtained by each algorithm.

**Fig 6:**
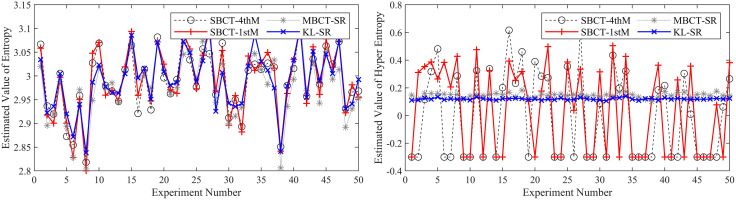
The estimated values of the *En* and *He* for CMKP (25, 3, 0.1).

**Fig 7:**
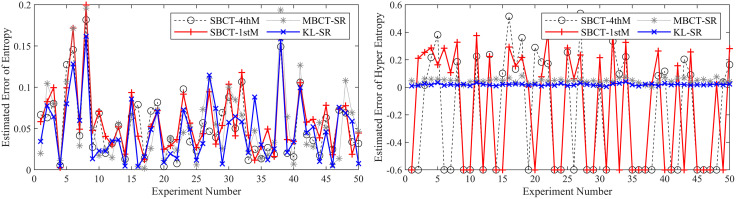
The estimation errors of the *En* and *He* for CMKP (25, 3, 0.1).

**Fig 8:**
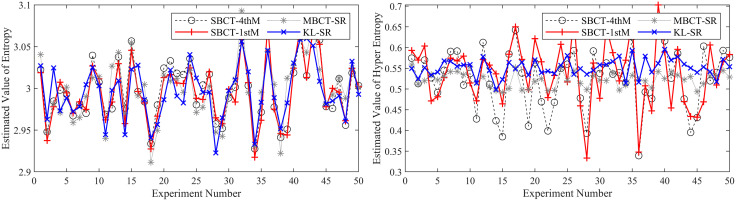
The estimated values of the *En* and *He* for CMKP (25, 3, 0.55).

**Fig 9:**
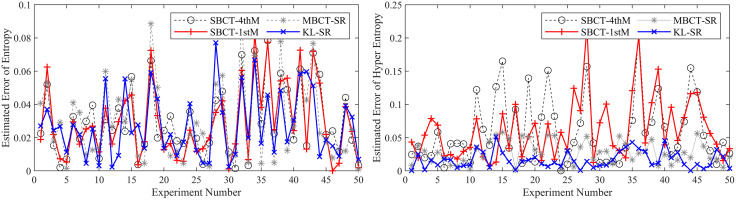
The estimation errors of the *En* and *He* for CMKP (25, 3, 0.55).

**Fig 10:**
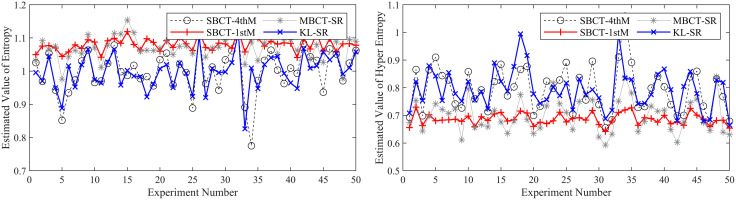
The estimated values of the *En* and *He* for CMKP (25, 1, 0.80).

**Fig 11:**
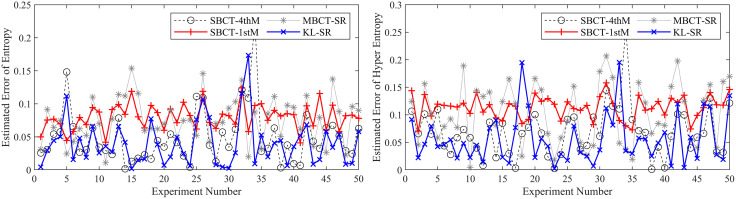
The estimation errors of the *En* and *He* for CMKP (25, 1, 0.80).

It is worth noting that due to the inherent limitations of the SBCT-4thM and SBCT-1thM algorithms, the CMKP obtained by transforming the modeling data may exhibit complex number phenomena. To distinguish them from other algorithms, we set a value of −0.3 for CMKP that are estimated to be complex numbers; and set the corresponding estimation errors to −0.6. When calculating the mean, mean absolute error, and mean square error, we selectively discard the complex data generated by the SBCT-4thM and SBCT-1thM transformations and only retain the data that has been transformed into real numbers. In addition, since the four algorithms have the same estimation method for the *Ex*, the mean, AAE, and MSE of the parameter estimation are consistent. Therefore, we don’t conduct an error analysis on the *Ex* for CM. Figs 6–11 show in detail the key parameter estimation results of the cloud model for three datasets (*D*_1_, *D*_2_, *D*_3_). Tables 4–6 present the mean, AAE and MSE of parameter estimates for four algorithms.

**Table 4:** The estimation results of CMKP (25, 3, 0.1).

CMKP	Mean, AAE, MSE	SBCT-4thM	SBCT-1thM	MBCT-SR	KL-SR
*En*	Mean	2.9888	2.9903	2.9798	**2.9911**
	AAE	0.0535	0.0599	0.0539	**0.0483**
	MSE	0.0044	0.0052	0.0050	**0.0037**
*He*	Mean	0.2956	0.3123	0.1479	**0.1195**
	AAE	0.3034	0.3021	0.0479	**0.0195**
	MSE	0.1105	0.1061	0.0025	**0.0004**

**Table 5:** The estimation results of CMKP (25, 3, 0.55).

CMKP	Mean, AAE, MSE	SBCT-4thM	SBCT-1thM	MBCT-SR	KL-SR
*En*	Mean	**2.9987**	2.9961	2.9979	2.9964
	AAE	0.0301	0.0291	0.0296	**0.0271**
	MSE	0.0013	0.0013	0.0014	**0.0011**
*He*	Mean	0.5242	0.5386	0.5227	**0.5514**
	AAE	0.0601	0.0624	0.0277	**0.0166**
	MSE	0.0061	0.0060	0.0010	**0.0004**

**Table 6:** The estimation results of CMKP (25, 1, 0.8).

CMKP	Mean, AAE, MSE	SBCT-4thM	SBCT-1thM	MBCT-SR	KL-SR
*En*	Mean	**0.9994**	1.0800	1.0740	0.9985
	AAE	0.0490	0.0800	0.0758	**0.0401**
	MSE	0.0040	0.0067	0.0068	**0.0027**
*He*	Mean	0.7930	0.6865	0.7002	**0.7942**
	AAE	0.0685	0.1134	0.1036	**0.0561**
	MSE	0.0067	0.0132	0.0136	**0.0051**

Based on the above experimental results, the analysis is as follows:

1. From Figs 6 and 7, when the modeling data is in a relatively aggregated non-atomization state, namely the *He* of CM is less than *En*/3. In this case, if *He* is too small relative to *En*, complex number cases may occur when obtaining estimates of *He* based on the SBCT-4thM and SBCT-1thM algorithms. Specifically, the SBCT-4thM algorithm generated complex numbers 25 times in 50 BCT experiments, while the SBCT-1thM algorithm generated complex values 23 times. This indicates that these two algorithms cannot obtain ideal CMKP when processing such data. In addition, from the experimental results that did not generate complex numbers, it can be seen that these two algorithms have significant fluctuations in their estimation of *He*, indicating that their estimation of *He* is not stable enough in this specific situation. At the same time, the MBCT-SR and KL-SR algorithms do not exhibit complex numbers for *He* under the same data input conditions, and according to the experimental results presented in Table 4, the KL-SR algorithm shows significantly lower errors in estimating the *En* and *He* compared to the other three algorithms.
2. From Fig 8, as the *He* value increases, although the SBCT-4thM and SBCT-1thM algorithms no longer exhibit complex number cases, their estimates of *En* and *He* still fluctuate greatly. During the process of 50 BCT experiments, the fluctuation amplitude of these two algorithms is significantly greater than that of MBCT-SR and KL-SR algorithms, especially when estimating *He*. In addition, according to Fig 9 and the results in Table 5, the KL-SR algorithm does not cause significant fluctuations in the estimated values of the *En* and *He* due to changes in *He*, and its accuracy is also higher than other algorithms. This indicates that the KL-SR algorithm exhibits better stability and accuracy when processing non-atomization data.
3. From Figs 10 and 11, when the data is in a relatively dispersed atomization state, i.e., the *He* of CM is greater than *En*/3. The SBCT-1thM and MBCT-SR algorithms showed significant deviations in estimating *He*, with their estimated values significantly different from the actual parameter of 0.80. Based on the experimental results in Table 6, we can further observe that the estimation accuracy of the SBCT-4thM algorithm is not ideal under atomization conditions. In contrast, the KL-SR algorithm demonstrated higher accuracy in estimating *He*. It not only can more accurately approach the actual parameter values but also maintains high stability in multiple experiments.

### 4.2. Error analysis of BCT algorithm based on real data

In order to further verify the universality of the KL-SR algorithm, this paper conduct experiments using real fault data collected from the flexible rotor system of the ZHS-2 multifunctional motor rotor, as well as three publicly available datasets in the UCI database. The fault diagnosis dataset reflects the data information of the motor rotor in a normal state and three fault states (rotor imbalance, rotor misalignment, and rotor base looseness). The four features mainly include the vibration acceleration amplitudes from the 1st to the 3rd harmonic and the average amplitude of time-domain vibration displacement. In addition, the seed dataset, wine dataset, and iris dataset are selected from the UCI database. Among them, the seed dataset contains 7 features of 3 wheat varieties, the wine dataset records compound information of different wine varieties (such as malic acid and ash content), and the iris dataset contains morphological features for three types of iris flowers. The basic information of each dataset is listed in Table 7.

**Table 7:** Experimental dataset information.

Dataset	Attributes	Classes	Samples
Rotor	4	4	800
Seeds	7	3	210
Wine	13	3	178
Iris	4	3	150

We extract data from the motor rotor fault diagnosis dataset and the UCI dataset under the same features for analysis of different categories. Specifically, the average amplitude data of time-domain vibration displacement under three fault states are obtained from the fault diagnosis dataset, and are denoted as Rotor1, Rotor2, and Rotor3, respectively. Each type of data contains 50 samples. In addition, we sequentially extract width data of three wheat seed varieties from the seed dataset, label as Seeds1, Seeds2, and Seeds3; magnesium content data of three wine categories from the wine dataset, designate as Wine1, Wine2, and Wine3; and sepal length data of three iris species from the iris dataset, named Iris1, Iris2, and Iris3. These data are used as modeling data in the experiment to verify the effectiveness of the algorithm.

**Fig 12:**
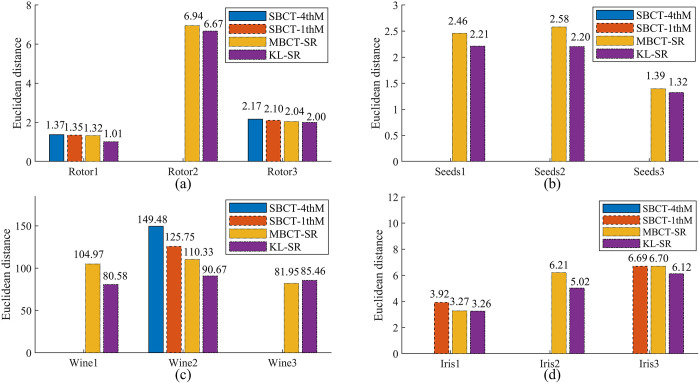
The results of Euclidean distance for the experimental dataset.

For each extracted dataset, we sequentially used four BCT algorithms to obtain the CMKP. Table 8 present CMKPs corresponding to the aforementioned four sets of modeling data. Considering the interrelationship between FCT and BCT of CM, we further generate corresponding estimation data using the FCT method based on the obtained CMKP. The accuracy of our proposed method is assessed by calculating the Euclidean distance between the original real data (modeling data) and the estimated data post-transformation. For the case of complex number estimation of *He* using SBCT-4thM and SBCT-1thM, we set the Euclidean distance between them and the real data to *NaN* for subsequent accuracy comparison. Figs 12a–12d show the Euclidean distances before and after the transformation for four BCT methods on four different types of datasets, respectively.

**Table 8:** The CMKP of experimental dataset.

Dataset		SBCT-4thM	SBCT-1thM	MBCT-SR	KL-SR
Rotor	Rotor1	(4.3455, 0.0625, 0.0257)	(4.3455, 0.0657, 0.0158)	(4.3455, 0.0673, 0.0030)	(4.3455, 0.0685, 0.0033)
	Rotor2	**(4.6952, 0.3107, 0.0699i)**	**(4.6952, 0.3108, 0.0707i)**	(4.6952, 0.3017, 0.0084)	(4.6952, 0.3035, 0.0108)
	Rotor3	(9.8097, 0.1000, 0.0266)	(9.8097, 0.1026, 0.0130)	(9.8097, 0.1033, 0.0039)	(9.8097, 0.1041, 0.0050)
Seeds	Seeds1	**(3.2446, 0.1808, 0.0338i)**	**(3.2446, 0.1819, 0.0394i)**	(3.2446, 0.1761, 0.0054)	(3.2446, 0.1751, 0.0070)
	Seeds2	**(3.6774, 0.1895, 0.0387i)**	**(3.6774, 0.1917, 0.0483i)**	(3.6774, 0.1843, 0.0052)	(3.6774, 0.1837, 0.0064)
	Seeds3	**(2.8537, 0.1511, 0.0330i)**	**(2.8537, 0.1530, 0.0408i)**	(2.8537, 0.1464, 0.0041)	(2.8537, 0.1486, 0.0055)
Wine	Wine1	**(105.6250, 9.9466, 1.5942i)**	**(105.6250, 9.8855, 1.1529i)**	(105.6250, 9.6788, 0.3029)	(105.6250, 9.6862, 1.7002)
	Wine2	(94.4250, 10.9992, 14.4660)	(94.4250, 14.7562, 10.6068)	(94.4250, 17.8704, 1.1293)	(94.4250, 17.8796, 21.4347)
	Wine3	**(99.9750, 11.0110, 2.4676i)**	**(99.9750, 11.0573, 2.6668i)**	(99.9750, 10.5995, 0.2971)	(99.9750, 10.5865, 2.4689)
Iris	Iris1	**(5.0060, 0.3582, 0.0637i)**	(5.0060, 0.3392, 0.0955)	(5.0060, 0.3486, 0.0109)	(5.0060, 0.3484, 0.0657)
	Iris2	**(5.9360, 0.5295, 0.1182i)**	**(5.9360, 0.5281, 0.1120i)**	(5.9360, 0.5105, 0.0141)	(5.9360, 0.5098, 0.0179)
	Iris3	**(6.5880, 0.6397, 0.0699i)**	(6.5880, 0.6298, 0.0872)	(6.5880, 0.6297, 0.0206)	(6.5880, 0.6267, 0.0239)

The complex number values are in bold.

The results of solving cloud model feature parameters (CMKP) for each dataset presented in Table 8 under four algorithms can be analyzed in depth from multiple dimensions:

1. The SBCT-4thM and SBCT-1thM algorithms have shown similar performance in solving key parameters of cloud models. Both algorithms output complex solutions containing imaginary units in multiple subsets of the rotor, seed, wine, and iris datasets. Among them, the Seeds1, Seeds2, and Seeds3 subsets of the seed dataset all present complex *He* parameters. From the perspective of algorithm principles, the core flaw of these two traditional backward cloud transformation conversion methods is that they do not consider the heterogeneity of data distribution. It adopts a single parameter fitting method, which is prone to negative variance terms in the mathematical solution process of backward cloud transformation when dealing with atomized datasets with nonlinear and non-Gaussian distribution characteristics. Ultimately, this resulted in the inability to extract effective cloud model feature parameters, directly losing the BCT algorithm’s ability to represent data features.
2. The MBCT-SR algorithm successfully avoids the problem of complex solutions by implementing a strategy of repeated sample extraction. However, from the numerical results, although the parameter estimation values of MBCT-SR algorithm are real numbers, they show deviations from the true data distribution in some sub datasets. The root cause lies in the algorithm’s ensemble modeling strategy, which fails to fully consider the heterogeneity between data with different degrees of atomization. Due to the lack of adaptive modeling mechanisms for different data distribution characteristics, the robustness of parameter estimation is ultimately insufficient.
3. For the KL-SR algorithm we proposed, it not only completely avoids the complex solution problem, but also exhibits better BCT feature representation performance, which is due to its dual state adaptation design that integrates non-atomized state and atomized state. From the perspective of data processing, the KL-SR algorithm measures the distribution similarity between different atomized sample sets and the original data through KL divergence, adaptively selects the optimal atomized sample size and fitting strategy, and avoids the negative variance term caused by data distribution mismatch in backward cloud transformation from the source; From the perspective of parameter estimation, this algorithm introduces parameter estimation and kurtosis calculation for correction in CMKP solution under atomization state, ensuring the real number and physical meaning rationality of core parameters such as *He*. The parameter estimation values of KL-SR algorithm on various datasets are of the same order of magnitude as MBCT-SR, and it exhibits numerical results that are closer to the true characteristics of data in complex distribution datasets such as seeds and irises, verifying its effectiveness and applicability on real datasets.

Moreover, as evident from Fig 12, both the KL-SR and MBCT-SR algorithms maintain the validity after BCT for four different types of data. Notably, the effectiveness is more pronounced on the Seeds dataset, as depicted in Fig 12b. Meanwhile, the Euclidean distance of KL-SR on each dataset is smaller than the other three algorithms, indicating that the method of introducing KL divergence to measure data distribution can effectively improve the accuracy of BCT. These experimental results confirm the effectiveness and reliability of the proposed KL-SR method.

### 4.3. Cost analysis of backward cloud transformation algorithm

The computational cost of the algorithm is one of the core indicators for evaluating the practicality of backward cloud transformation algorithm engineering. Its quantitative analysis needs to be combined with theoretical derivation of time complexity and experimental verification of actual running time, while balancing the relationship between cost and algorithm accuracy. This section analyzes the computational costs of four backward cloud transformation conversion methods, SBCT-4thM, SBCT-1stM, MBCT-SR, and KL-SR, from three dimensions: theoretical complexity, experimental validation, and cost-effectiveness trade-off.

#### 4.3.1. Theoretical complexity analysis of algorithm cost.

As a core indicator for measuring the computational cost of algorithms, time complexity essentially characterizes the dependency relationship between algorithm running time and input scale (number of cloud drops *N*), core parameters (sampling frequency, sample size, etc.). The time complexity of the four algorithms is derived as follows:

1. SBCT-4thM and SBCT-1stM algorithms

Both algorithms are based on statistical moment estimation to implement backward cloud transformation, with the core operation being a single traversal of cloud drop data to calculate mean, variance, and moment statistics. All core operations are linear traversal without nested loops or complex sorting operations; therefore, the time complexity is *O*(*N*), which belongs to the linear cost algorithm. The computational cost is only positively correlated with the number of cloud drops *N*, making it the two most efficient algorithms in theory.

2. MBCT-SR algorithm

This algorithm introduces a repeated sampling mechanism, which requires completing *n* independent samplings and extracting *m* samples from *N* cloud drops each time to calculate the sample variance. The basic complexity of the algorithm is *O*(*N*) for linear traversal, while the core cost comes from *O*(*nm*) for sampling cycles, so the total time complexity is *O*(*N* + *nm*). When the sampling parameters satisfy *nm*>>*N*, the sampling cycle becomes the cost dominant term, and the actual complexity of the algorithm is determined by *O*(*nm*). The computational cost is significantly higher than the first two linear algorithms.

3. KL-SR algorithm

The computational cost of this algorithm consists of three key steps:① Sort cloud drops data in ascending order, with a time complexity of *O*(*N*log*N*) using classical sorting algorithms; ② Split the sorted data into two sub arrays and calculate KL divergence, which relies on data traversal and probability distribution estimation, with a complexity of *O*(*N*); ③ Multiple rounds of sampling and statistical calculation, completing *S* cycles and processing *n*_*s*_ samples each time, with a complexity of *O*(*Sn*_*s*_)=*O*(*N*). Overall, the *O*(*N*log*N*) of the sorting operation is the dominant complexity of the algorithm, while the linear complexity of the remaining steps can be ignored. Therefore, the total time complexity of the KL-SR algorithm is *O*(*N*log*N*), theoretically higher than the linear complexity of SBCT-4thM, SBCT-1stM. When the product of sampling parameters in MBCT-SR algorithm is too large, its complexity is higher than that of KL-SR algorithm.

#### 4.3.2. Experimental verification of algorithm running time.

To verify the conclusions of the theoretical complexity analysis mentioned above, this section uses 5000 cloud drops generated by three different sets of numerical features (CMKP) in Section 4.1 as inputs. The four algorithms are independently run 50 times, and the average running time is taken. The results are shown in Table 9 Meanwhile, the relative cost is calculated based on the running time of SBCT-1stM to further quantify the cost differences of each algorithm.

**Table 9:** Average running time and relative cost of four algorithms under different CMKPs.

CMKP	Algorithm Name	Average Running Time (s)	Relative Cost (Normalized to 1thM)
(25,3,0.10)	SBCT-4thM	9.826 × 10−4	4.71
	SBCT-1thM	2.084 × 10−4	1.00
	MBCT-SR	2.220 × 10−2	106.53
	**KL-SR**	**7.100 × 10−3**	**34.07**
(25,3,0.55)	SBCT-4thM	9.898 × 10−4	4.63
	SBCT-1thM	2.136 × 10−4	1.00
	MBCT-SR	1.260 × 10−2	59.00
	**KL-SR**	**7.000 × 10−3**	**32.77**
(25,1,0.80)	SBCT-4thM	5.902 × 10−4	5.06
	SBCT-1thM	1.167 × 10−4	1.00
	MBCT-SR	6.700 × 10−3	57.41
	**KL-SR**	**4.200 × 10−3**	**35.99**

The following conclusions can be drawn from the experimental results:

1. Compared to the cost of linear algorithms: The running time of MBCT-SR and KL-SR are both in the range of 10^−3^ ~ 10^−2^, with relative costs of 32.77 ~ 106.53 and 34.07 ~ 59.00, respectively, which are significantly higher than linear algorithms. The core reason is that the nested operations introduced by the sampling cycle greatly increase the computational overhead.
2. The cost difference between KL-SR and MBCT-SR: The average running time of KL-SR is shorter than that of MBCT-SR, because MBCT-SR uses a higher number of sampling groups and cloud drops per group, that is, the product of sampling parameters is much larger than the number of cloud drops. At this point, the actual computational cost of the sampling cycle far exceeds the cost of sorting operations in KL-SR, resulting in MBCT-SR having a longer actual running time than the KL-SR algorithm.

#### 4.3.3. Cost performance trade-off discussion.

Cost analysis shows that although the computational cost of KL-SR is higher than SBCT-4thM and SBCT-1stM, it evaluates the atomization state of data through KL divergence, significantly reducing the estimation errors of *En* and *He*. In the experiment, the *En* and *He* errors of KL-SR were much lower than those of the two types of linear moment estimation algorithms. At the same time, the sampling parameters of KL-SR are better than MBCT-SR, and the actual running time is slightly shorter. In the scenario of offline cloud model training and high-precision digital feature estimation, its accuracy advantage can compensate for the lack of computational cost and has significant application value.

## 5. Conclusion

To address the inherent limitation of existing backward cloud transformation (BCT) algorithms—neglecting the distribution characteristics of sample data during the extraction of cloud model key parameters (CMKPs, i.e., expectation *Ex*, entropy *En*, and hyper entropy *He*)—this study proposes a KL divergence-based BCT algorithm. The primary contribution of this work lies in the development of a refined CMKP extraction framework integrated with data distribution analysis: coarse-grained atomization estimation is first performed on sample data, followed by the construction of an atomization template dataset. The atomization state of modeling data is quantified through KL divergence-based comparison, and two differentiated strategies are designed accordingly: the Non-Atomization Transformation Strategy for low-uncertainty and non-atomized data, and the Atomization Transformation Strategy for high-uncertainty and atomized data. This tailored framework ensures the accuracy of CMKP extraction across diverse data distribution scenarios. Comparative experiments are conducted on the UCI benchmark datasets and real-world fault diagnosis data, with traditional BCT algorithms (including SBCT-1thM, SBCT-4thM, and MBCT-SR) as baselines. Experimental results demonstrate that the proposed algorithm outperforms existing methods in both the accuracy and stability of CMKP extraction. From a broader perspective, this study enriches the theoretical system of uncertainty modeling by explicitly incorporating data distribution differences into the BCT process. It provides a reliable technical reference for cloud model applications in fields with significant data heterogeneity, such as complex industrial fault diagnosis and dynamic system evaluation. In future research, efforts will be directed toward optimizing the algorithm under data loss conditions, aiming to further enhance its robustness and adaptability to complex practical application scenarios, thereby expanding the application scope and practical value of cloud model-based uncertainty processing.

## Supporting information

S1 File Sensitivity analysis of the atomization state threshold (*He* = *En*/3). (PDF)

S2 File Validation experiments on the initial number of cloud drops and template set size. (PDF)
