# Supplementary material for: Backward cloud transformation algorithm based on Kullback Leibler divergence
Source: PLoS One. 2026 Jan 27;21(1):e0341268. doi: 10.1371/journal.pone.0341268 (PMC12843599; doi:10.1371/journal.pone.0341268)
Supplement: S1 File — (PDF) [file pone.0341268.s001.pdf]

Appendix

A: Sensitivity Analysis of the Atomization State Threshold (*He*=*En*/3)

In Section 3.2 of the main text, the theoretical rationale for setting the atomization state threshold to *He*=*En*/3 has been elaborated. To facilitate a clearer understanding of this parameter choice and to evaluate its impact on algorithm performance, this appendix provides a corresponding sensitivity analysis.

Specifically, we designed comparative experiments to quantitatively verify the optimal value of the atomization threshold. The experiment used subsets Wine1, Wine2, and Wine3 of the real sample Wine in Section 4.2 as the objects to be modeled and performed 50 independent backward cloud transformation experiments using the KL-SR algorithm. Five sets of atomization threshold variables were set up in the experiment, namely *He*=4*En*/3, *He*=2*En*/3, *He*=*En*/3, *He*=*En*/6, and *He*=*En*/12. By calculating the Euclidean distance between the modeling data and the estimated data obtained by algorithm transformation, the impact of different atomization thresholds on the accuracy of cloud model modeling was quantitatively analyzed. The experimental results are shown in Table 1. To facilitate the analysis of the impact of atomization threshold on transformation strategy, Table 1 synchronously provides the KL divergence values $KL(\tilde{D}_L \| \tilde{D}_H)$ of modeling data, KL divergence values $KL(\tilde{D}_L^{'} \| \tilde{D}_H^{'})$ of atomization template set, and the backward cloud transformation strategy (BCTS) determined based on KL divergence values: Non-Atomization Backward Cloud Transformation Strategy (NATS) and Atomization Backward Cloud Transformation Strategy (ATS).

Table 1. Estimation results of cloud model parameters under different atomization thresholds

Dataset	Threshold	$KL(\tilde{D}_L \| \tilde{D}_H)$	$KL(\tilde{D}_L^{'} \| \tilde{D}_H^{'})$	BCTS	Euclidean distance
	He=4*En*/3	3.2396	1.1422	NATS	80.58
	He=2*En*/3	3.2396	1.7133	NATS	80.58
Wine1	***He*=*En*/3**	**3.2396**	**1.9146**	**NATS**	**80.58**
	He=*En*/6	3.2396	2.5029	NATS	80.58
	He=*En*/12	3.2396	3.4439	ATS	86.24
	He=4*En*/3	1.9981	1.7757	NATS	146.60
	He=2*En*/3	1.9981	1.9975	NATS	146.60
Wine2	***He*=*En*/3**	**1.9981**	**3.9306**	**ATS**	**90.67**
	He=*En*/6	1.9981	4.1397	ATS	90.67
	He=*En*/12	1.9981	4.4252	ATS	90.67
	He=4*En*/3	2.3261	1.7398	NATS	96.64
	He=2*En*/3	2.3261	1.2309	NATS	96.64
Wine3	***He*=*En*/3**	**2.3261**	**2.5285**	**ATS**	**85.46**
	He=*En*/6	2.3261	3.8185	ATS	85.46
	He=*En*/12	2.3261	4.1374	ATS	85.46

According to the process described in Section 3.2 of the article, the value of the atomization threshold directly determines the transformation process selection of the KL-SR algorithm (Eq. (12)). Different thresholds correspond to differentiated transformation strategies, which in turn leads to differences in cloud model modeling results. Based on the experimental results in Table 1, it can be concluded that:

1) The atomization threshold indirectly affects the decision-making process of the KL-SR algorithm's backward cloud transformation strategy by regulating the KL divergence value of the atomization template set. For the Wine1, Wine2, and Wine3 datasets, as the atomization threshold decreases, the KL divergence values corresponding to the atomization template set show a monotonically increasing trend. Taking the Wine1 dataset as an example, as the atomization threshold changes, the KL divergence value of the atomization template set increases from 1.1422 to 3.4439.

2) For the Wine1 dataset, when the atomization threshold is set to *En*/12, BCTS is converted from NATS to ATS, and the transformed Euclidean distance changes from 80.58 to 86.24; For the Wine2 dataset, when the atomization threshold is set to 2*En*/3, BCTS is NATS and the Euclidean distance increases to 146.60; For the Wine3 dataset, when the atomization threshold is set to 2*En*/3, the Euclidean distance increases to 96.64. All three datasets have the minimum Euclidean distance when the atomization threshold is *En*/3.

Based on comprehensive theoretical analysis and experimental verification of the proposed method, it can be concluded that selecting *En*/3 as the atomization threshold for the KL-SR algorithm is the optimal solution that balances estimation accuracy and scene adaptability.
