# Supplementary material for: Backward cloud transformation algorithm based on Kullback Leibler divergence
Source: PLoS One. 2026 Jan 27;21(1):e0341268. doi: 10.1371/journal.pone.0341268 (PMC12843599; doi:10.1371/journal.pone.0341268)
Supplement: S2 File — (PDF) [file pone.0341268.s002.pdf]

Appendix

B: Validation Experiments on the Initial Number of Cloud Drops and Template Set Size

This appendix aims to experimentally validate the rationale, as stated in the main text, for setting both the initial number of input cloud drops and the size of the atomization template set to 5000.

To systematically validate the rationality of this choice, we conducted the following experiments. Specifically, based on a given set of cloud model characteristics CMKP = (25, 3, 0.1), generate cloud drops with quantities of 1000, 2000, ..., 10000 as algorithm inputs, and perform 50 independent backward cloud transformation experiments. The trend of the average estimation value and mean square error (MSE) of *He* under different sample sizes is shown in Fig 1.

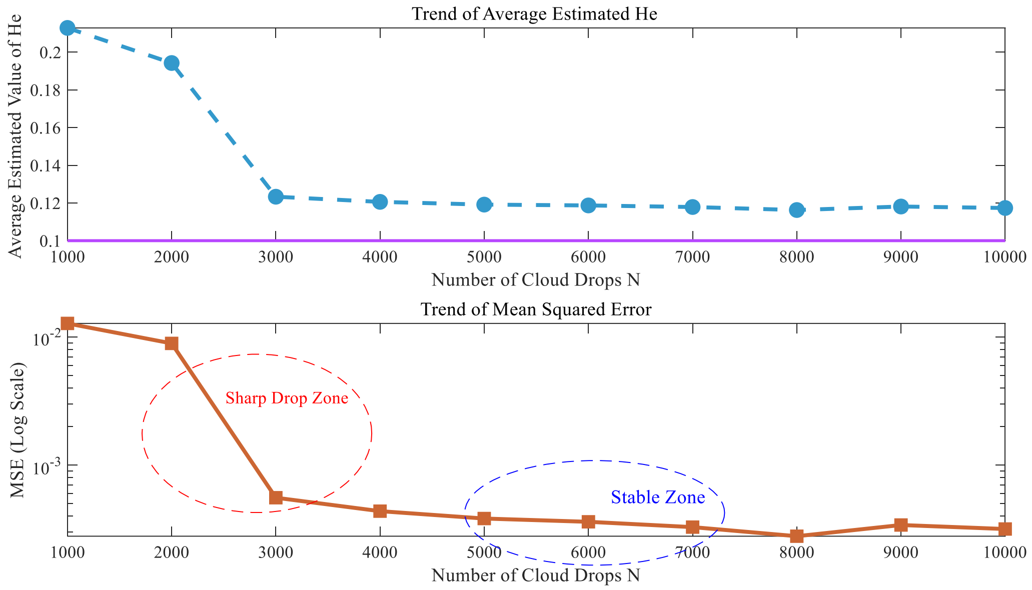

Fig 1. Variation trend of *He* with increasing cloud drops

According to Fig 1, as the number of cloud drops increases, when the number of cloud drops is in the range of 2000~3000, the parameter estimation error of KL-SR algorithm enters a sharp drop zone, and the deviation between the estimated *He* value and the true value continues to decrease with the increase of cloud drop number; When the number of cloud drops further increases to the range of 5000~7000, the estimation error of *He* shows a stable convergence characteristic. Although the continued increase in the number of cloud drops can slightly reduce the estimation error of *He*, it will significantly increase the time and computational cost of KL divergence calculation in the KL-SR algorithm. Therefore, this study selected the minimum number of cloud drops within the stable zone, i.e. *N*=5000, as the optimal input scale for the KL-SR algorithm.
